# Psychological Distress and Social Adjustment of a Working Adult Population with Single-Sided Deafness

**DOI:** 10.3390/audiolres14060091

**Published:** 2024-12-12

**Authors:** Enrico Apa, Riccardo Nocini, Andrea Ciorba, Luca Sacchetto, Chiara Gherpelli, Daniele Monzani, Silvia Palma

**Affiliations:** 1 Audiology Unit, Dipartimento di Scienze Cliniche e di Comunità, Dipartimento di Eccellenza 2023–2027, Fondazione IRCCS Ca’ Granda, Policlinico of Milan, 20122 Milan, Italy; enrico.apa@policlinico.mi.it; 2 Otorrhinolaryngology, Department of Surgical Sciences, Dentistry, Gynaecology and Paediatrics, University of Verona, Borgo Roma Hospital, 37134 Verona, Italy; riccardo.nocini@univr.it (R.N.); luca.sacchetto@univr.it (L.S.); daniele.monzani@univr.it (D.M.); 3 Otorhinolaryngology and Audiology Department, University Hospital, via Aldo Moro 8, Cona, 44124 Ferrara, Italy; andrea.ciorba@unife.it; 4 Otorhinolaryngology and Audiology Unit, Department of Medical and Surgical Sciences for Children and Adults, Azienda Ospedaliero-Universitaria of Modena, via del Pozzo 71, 41100 Modena, Italy; chiara.gherpelli@unimore.it; 5 Audiology, Primary Care Department, AUSL of Modena, 41100 Modena, Italy

**Keywords:** unilateral hearing loss, single side deafness, social functioning questionnaire, hearing handicap inventory for adult, working

## Abstract

**Background**: Hearing loss is a highly prevalent condition in the world population that determines emotional, social, and economic costs. In recent years, it has been definitely recognized that the lack of physiological binaural hearing causes alterations in the localization of sounds and reduced speech recognition in noise and reverberation. This study aims to explore the psycho-social profile of adult workers affected by single-sided deafness (SSD), without other major medical conditions and otological symptoms, through comparison to subjects with normal hearing. **Methods**: This is a cross-sectional, case-control study. Subjects aged between 24 and 65 years, all currently employed and affected by SSD, were enrolled. They were administered both disease-specific and psychometric tests, such as the Hearing Handicap Inventory for Adults (HHIA), the Profile Questionnaire for Rating Communicative Performance, the Psychological General Well-Being Index (PGWBI), and the Social Functioning Questionnaire (SFQ). **Results**: A total of 149 subjects (mean age = 49.9; SD ± 8.5) were enrolled in the period 2021–2023; 68 were males (45.6%), and 81 were females (54.4%). The normal hearing group was composed of 95 subjects, and the SSD sample was composed of 54 subjects. The results of our study show that the levels of psychological well-being and social functioning in subjects with SSD are statistically worse than in the group of subjects with normal hearing in most subscales. **Conclusions**: This study definitely outlined evidence for a significantly worse psychological health status and a poorer social attitude of working adults affected by SSD with respect to their normal-hearing counterparts. Understanding the impact of SSD on patients’ work environment suggests a multidisciplinary approach to such patients in order to increase their quality of life through adequate counseling, acceptance, and role modeling.

## 1. Introduction

Hearing loss is a highly prevalent condition in the world population, determining emotional, social, and economic costs [1]. It is a common notion that bilateral hearing impairment can worsen patients’ quality of life, while, for years, it was assumed that a “single ear” could provide adequate hearing function, neglecting unilateral deficit. The size of the affected population is being determined. The prevalence of unilateral hearing loss (UHL) in the US has been estimated at around 8% [2], and in other experiences, the percentages vary from 3.2 to 19.4% [3]. Etiology can be tied to genetic diseases, inner ear malformations, temporal bone fractures, Meniere’s disease, tumors, autoimmune disease, and ototoxic drugs; sudden hearing loss is one of the most frequent [4]. The entity can vary from mild to profound. The more severe cases refer to single-sided deafness (SSD), a condition with normal or near normal hearing (pure-tone average [PTA] of ≤25 dB) in one ear and hearing with PTA > 70 dB. The SSD is estimated at about 0.14% [5,6].

It has been definitely recognized that the lack of physiological binaural hearing (the so-called “binaural advantage”) causes alterations in the localization of sounds, worse frequency selectivity, reduced speech recognition in noise and reverberation, and a subjective sensation of reduced sound intensity [7,8,9]. Furthermore, an increased effort to compensate for SSD in complex listening acoustic scenarios has been documented [10]. Over time, such additional stressors might result in auditory fatigue and reduced performance at work from the extra effort that listening with one ear requires [11,12,13]. The consequences of unilateral hearing impairment in children were first investigated by Bess and Tarpe in 1984 [14]. Since then, several studies have been carried out to explore childhood’s psychosocial and psychoeducational consequences. On the contrary, few attempts are made in the medical literature to investigate psychosocial attitudes in adults suffering from this condition. Anyway, the results of these studies seem not to be fully definitive because of the very small sample size [15], the use of a single hearing disability questionnaire [16,17], the enrollment of subjects also affected by tinnitus and vertigo that could account for psychological distress per se [18], and above all, the lack of a control group composed of normal-hearing subjects.

Rehabilitation of unilateral hearing loss conditions is still a challenge, depending also on psychological factors. It happens frequently that many people delay seeking help until their hearing impairment severely impacts their life, mainly due to social stigma and a general reluctance to accept hearing loss. Recently, SSD has received an indication of the benefit of cochlear implants [7], as it has been shown to improve the quality of life for these patients. In this context, an analysis of the psychological profile of the subjects, as well as their expectations, is important.

This study aims to explore the psycho-social profile of adult workers affected by SSD without other major medical conditions and otological symptoms in comparison to subjects with normal hearing. Due to the objective of this study, patients were evaluated before any hearing rehabilitation.

Both disease-specific and psychometric tests were administered to both groups.

## 2. Materials and Methods

This is a cross-sectional, case-control study. The subjects were outpatients of the Audiology service of the University Hospitals of Modena in the years 2021–2023. This study is a preliminary report from a larger investigation on hearing disability and handicap in adult workers conducted in accordance with the Declaration of Helsinki and approved by the Ethics Committee of Modena-AVEN (protocol code AOU 0015022/21, 11 May 2021) for studies involving humans. All the subjects involved in this study signed an informed consent.

Inclusion criteria were age between 24 and 65 years (working age) and attendance at work activities. SSD cases were enrolled if 4fPTA ≥ 70 dB HL [6] and ≤25 dB in the better ear.

Exclusion criteria were the presence of fluctuating hearing loss, tinnitus, and/or vertigo; syndromes, ear malformations, neurological and psychiatric diseases; and therapy with psychotropic drugs such as antidepressants, anxiolytics, and antipsychotics. Also, major medical conditions, such as tumors and cardiovascular and metabolic diseases that could account per se for a self-perceived disability, were the causes of exclusion of both patients and controls from the casuistry. Enrollment criteria were strict to reduce bias, and all subjects selected did not wear auditory devices, such as hearing aids, or were not cochlear implant users when the present audiological evaluation was ongoing.

Patients underwent audiological evaluation, including medical history collection, otoscopy, tympanometry, and pure tone audiometry using air and bone conduction in accordance with the British Society of Audiology recommended procedures (British Society of Audiology 2012). After determining the threshold for all frequencies, the average threshold for each ear, 500, 1000, 2000, and 4000 Hz (4fPTA = 4 frequency Pure Tone Average threshold), was used for statistics.

According to the audiometric assessment, subjects were divided into two groups: the normal hearing group (NH) if 4PTA ≤ 25 dB and the SSD group.

Questionnaires

Subjects enrolled were then invited to complete the following PROMs (patient-reported outcome measures):

Hearing Handicap Inventory for Adults (HHIA) [19], Italian validated version [20] This questionnaire evaluates the perceived handicap by adults with hearing loss. It comprises 25 items: 13 focused on emotional consequences (emotional subscale) and 12 on social-situational consequences (socio-situational subscale). Each question has three possible answers that are associated with different scores: “no” corresponds to 0, “sometimes” corresponds to 2 points, and “yes” corresponds to 4 points. The emotional scale can vary from 0 to 52 points, and the social-situational can be from 0 to 48 points. The total score is included in the range of 0–100. The higher the score, the higher the perceived handicap.

Profile Questionnaire for Rating Communicative Performance [21], Italian version [22] This instrument, first presented by Sanders in 1950 and since then also termed Sanders’ Test, detects problems experienced by subjects in specific situations and different listening environment conditions. This 21-item survey evaluates the perceived difficulties of hearing and communicating of the subject in various situations at home (8 items), work (6 items), and social life (7 items). A possible answer is no difficulty (+2), little difficulty (+1), moderate difficulty (−1), and great difficulty (−2). The patients are also asked to rate the frequency with which the given situation is found: rarely (1), often (2), and very often (3). The score of any item is given by the multiplication between the level of difficulty and frequency. Negative scores indicate greater disability.

Psychological General Well-Being Index (PGWBI) [23] Italian version [24]. This questionnaire evaluates subjective perceptions of psychological well-being. It can be used to calculate psychological distress in different populations and for different diseases. The final validated version includes 22 items: 16 are questions, and 6 are affirmations. The instrument explores six domains: anxiety, depression, positivity and well-being, self-control, general state health, and vitality. Each domain is investigated using three to five items: in particular, five items concerning anxiety (25 points maximum), four items for positivity, well-being, and vitality (each 20 points maximum), and three items for depression, self-control, and general health (each 15 points maximum). Six possible answers are of close type and differentiated according to the item. Answers are posed in decreasing order of score, from the most positive to the most negative or vice versa; the intensity and frequency of the phenomenon are investigated. Based on the answer, a score from 0 to 5 points can be assigned. The total score is included in the range of 0–110; the higher the score, the higher the psychological well-being. All items are referred to the four weeks before administration of the questionnaire.

Social Functioning Questionnaire (SFQ) [25]. Italian version [26]. This instrument evaluates perceived social functioning. It consists of eight items concerning essential aspects of social life, work, domestic tasks, financial worries, relationships in the family, sexual activity, social contacts, and free-time activities. Each answer can correspond to a score from 0 to 3 points: the higher the score, the worse the social functioning perceived. The sum of each score can vary between 0 and 24.

Statistical analysis

To provide a socio-demographic description of NH and UHL groups, we used a preliminary Pearson’s chi-square test to compare the distribution of categorical variables, such as gender (males and females), occupational (professional and non-professional), and social status (single and married or cohabiting). Continuous variables, such as age, duration of education (years), 4fPTA (dB HL), and questionnaire scores, were compared by the independent *t*-test procedure to test the null hypothesis that the means from the two samples are equal. Data analysis was performed using the Statistical Package for the Social Sciences version 25.0 (SPSS) (Chicago, IL, USA), and the statistically significant level was set at *p*-value < 0.05 in all procedures.

## 3. Results

A total of 149 subjects aged between 24 and 65 years (mean age = 49.9; SD ± 8.5) were enrolled in the period 2021–2023; 68 were males (45.6%), and 81 were females (54.4%). NH group is composed of 95 subjects, and SSD is composed of 54 subjects. Their clinical-social features are reported in Table 1. Concerning etiology, 27 (50%) cases had sudden onset, 12 (22%) were of viral origin (mumps, etc.), and 15 (27%) remained unidentified as the clinical history was not explanatory. Sudden hearing loss cases were enrolled when the diagnostic-therapeutic process was concluded, and the hearing threshold was stabilized; moreover, many subjects presented long-lost hearing loss and were stable over time, having not accepted a rehabilitation program at the time of examination.

Most of the subjects in both groups were living in a family, and a slight prevalence of graduates and those employed in a professional job was found in the NH group. No difference reached a statistically significant level.

**Figure 1:**
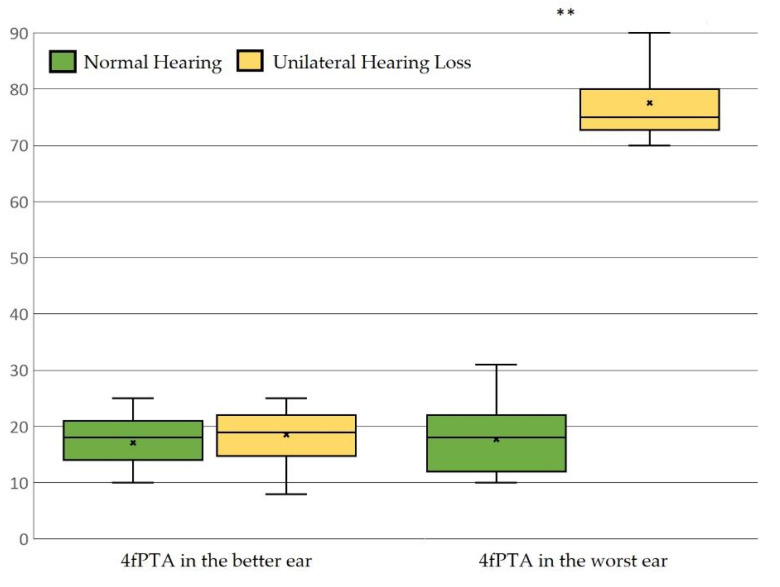
4fPTA in the better and worse ear in the two groups. Each box is included between the first and third quartile; the box’s height is equivalent to the inter-quartile range (IQR) and contains 50% of the measurements. Since no values deviated from the box by more than 1.5 of IQR upwards or downwards, no potential outliers were observed. The Independent *t*-test was used for statistical analysis. * *p*-value < 0.05, ** *p*-value < 0.005.

Hearing thresholds of the subjects are reported in Figure 1. As shown, in NH and SSD groups, the 4fPTA, on the better side, was 17.15 dB HL (10–20 dB HL; SD: ±4.79 dB HL) and 18.52 dB HL (8–25 dB HL; SD: ±4.60 dB HL), respectively (*p* = 0.090). The 4fPTA, on the other side, was 17.69 dB (10–31 dB; SD: ±5.82 dB) in the NH group and 77.54 dB (70–100 dB; SD: ±6.76 dB) in the SSD group (*p* < 0.0001). 4fPTA is the better and worse ear in the two groups.

Significant differences between the two groups were observed in both total scores and in all subscales of the Sanders’ test (ST) and HHIA in the two groups. (Table 2). In particular, higher values for the HHIA and lower values for the ST were detected in the SSD group.

Significantly, higher PGWBI scores were observed in the overall instrument and in the subdomains Depressed Mood, Positive Well-being, General Health, and Vitality (Table 3), while in Anxiety and Self-Control, the differences were not significant. Table 4 shows the scores of the SFQ, which clearly indicate a porter social adjustment in patients with respect to controls.

## 4. Discussion

The results of our study confirm that the levels of psychological well-being and social functioning of subjects with SSD are statistically worse than those of subjects with no hearing loss.

Both the HHIA emotional and socio-situational subscales scores were statistically different in the SSD group and underpinned more personal difficulties in daily activities, such as participating in social events and going to the theater, cinema, etc. An emotional state of irritability and frustration, deflection of mood, and perception of loneliness were detected.

An increase in relationship problems with family and friends is also reported in small sample studies [27]. Interestingly, Chang et al. [28] identified the increasing level of dependence from family members and a poor sense of self-efficacy as determining moodiness in SSD patients.

The difficulties in social participation and interpersonal relationships are confirmed by the SFQ, which has been revealed to be a robust instrument to assess social functioning in essential aspects, such as work tasks, relationships with family, and social contacts [25].

Concern about difficulties in the working environment emerged. Employers with hearing loss express higher levels of stress, low levels of psychological well-being, and worse health states [29]. These subjects reveal high levels of tiredness in working and are often absent due to illness [30,31]. These findings are consistently replicated by our results in SSD patients.

The PGWBI scores of the Depression, Vitality, Health, Positivity, and Well-being subscales results are all statistically worse in SSD subjects; findings concerning the Vitality subscale suggest hearing fatigue, as demonstrated in children with SSD [11]. Subjects with unilateral deafness experience a significant disability in auditory function that affects their communication and social interaction [32]. On the contrary, in subscales that measure anxiety and self-control, the two groups answered with similar scores. This can be attributed to the self-perception of SSD condition as a disturbing but not completely invalidating disease that may be due to many different psychological traits, such as copying strategy and locus of control, that were not assessed in this study [33].

In the Blue Mountain Hearing Study, scores concerning disability perceived by these subjects through the 36-item Short-Form Health Survey are similar to those of the normal hearing population [34]. A wide range of methodological variations, such as inclusion age, around 67 years, disability/handicap, and social adjustment scales, may underpin this discrepancy with the results of our study.

In a previous study, we found that working adults with mild to moderate sensorineural hearing loss experience more negative emotional reactions and socio-situational limitations than subjects with no hearing problems and that deterioration of health-related quality of life in these specific domains would occur [35].

In a recent study [15], interviews were conducted using the critical incident technique, and a range of functional hearing difficulties associated with SSD were reported to affect social and psychological well-being. Subjects interviewed reported also worrying about losing the hearing in their other ear and embarrassment related to the social stigma attached to hearing loss. In this scenario, traditional questionnaires, both disease-specific and psychometric, could be flanked by more structured psychological evaluations in order to implement a tailored treatment based not only on the hearing threshold but also on each subject’s mental health status. In particular, this study suggested that most controls (NH group) were females with high-grade instruction and professionals, but no further investigation was performed because of a sample size that was too limited for multifactorial statistical analyses.

A further limitation of this study is the lack of a preliminary psychiatric evaluation that could confirm our results or identify abnormal personality traits.

On the contrary, the strengths of this study are exclusion criteria, such as tinnitus, vertigo, and major medical conditions that could be confounding cues per se when examining psychological distress and social adjustment.

## 5. Conclusions

Single-sided deafness is a condition that can chronically worsen the quality of life of affected subjects. Psychometric tests are important to reveal and quantify the consequence of this impairment on personal well-being. Understanding the impact of SSD on patients’ work environments suggests a multidisciplinary approach to such patients in order to increase their quality of life through adequate counseling, acceptance and confirmation, and role modeling. Moreover, these aspects should be specifically taken into account in working environments in terms of sick leave and the need for audiological rehabilitation. Therefore, this study provides evidence for a better understanding of single-sided deaf patients’ disability at work and for health professionals to provide a holistic approach to current and future SDD patients.

## Figures and Tables

**Table 1:** Personal data of the subjects of the study. Abbreviations: NH: Normal Hearing, SSD: Single Sided Deafness; SD: Standard Deviations; ^a^ Independent *t*-test, ^b^ Pearson’s chi-square test; * *p*-value < 0.05, ** *p*-value < 0.005.

Number of Cases	NH (95)	SSD (54)	Significance ^a,b^
Age	49.96	49.72	0.482 ^a^
	(24–65; SD: ±8.3)	(25–63; SD: ±8.2)	
Gender	54 females (56.8%)	27 females (50%)	0.420 ^b^
	41 males (43.2%)	27 males (50%)	
Familiar Status	34 married (35.8%)	13 married (24.1%)	0.139 ^b^
	61 in family (64.2%)	41 in family (75.9%)	
Educational qualification	2 primary schools (2.1%)	1 primary school (1.8%)	0.268 ^b^
	18 secondary schools (18.9%)	11 secondary schools (20.4%)	
	40 high schools (42.1%)	32 high schools (59.3%)	
	35 graduated (36.9%)	10 graduated (18.5%)	
Occupation	29 manual (30.5%)	24 manual (44.4%)	0.121 ^b^
	66 professional (69.5%)	30 professional (55.6%)	

**Table 2:** HHIA and ST results. Abbreviations: NH: Normal Hearing, SSD: Single-Sided Deafness; SD: Standard Deviations; HHIA: Hearing Handicap Inventory for Adults; ST: Sanders’s Test; ^a^ Independent *t*-test; * *p*-value < 0.05, ** *p*-value < 0.005.

Number of cases	NH (95)	SSD (54)	Significance ^a^
HHIA			
Total	13.71 (0–86; SD: ±19.78)	39.56 (0–74; SD: ±19.98)	0.000 **
Socio-Situational	5.35 (0–40; SD: ±8.83)	16.41 (0–34; SD: ±8.71)	0.000 **
Emotional	8.48 (0–48; SD: ±11.31)	23.26 (0–42; SD: ±11.57)	0.000 **
ST			
Total	79.23 (−13–132; SD: ±37.01)	13.04 (−50–120; SD: ±45.53)	0.000 **
Home Environment	33.04 (−8–54; SD: ±15.57)	5.65 (−27–49; SD: ±18.00)	0.000 **
Occupational Environment	21.25 (−5–36; SD: ±10.73)	3.13 (−36–34; SD: ±16.08)	0.000 **
Social Environment	24.94 (0–36; SD: ±12.33)	4.35 (−28–39; SD: ±14.89)	0.000 **

**Table 3:** PGWBI results. Abbreviations: PGWBI: Psychological General Well-Being Index; SD: Standard Deviations; ^a^ Independent *t*-test; * *p*-value < 0.05, ** *p*-value < 0.005.

Number of Cases	NH (95)	SSD (54)	Significance ^a^
**PGWBI**			
Total	81.42 (24–110; SD: ±14.20)	74.22 (58–98; SD: ±11.41)	0.002 **
Anxiety	18.03 (3–25; SD: ±4.03)	17.17 (9–24; SD: ±3.37)	0.184
Depressed Mood	13.38 (7–15; SD: ±1.56)	12.35 (8–15; SD: ±1.68)	0.000 **
Positive Well-being	12.57 (2–20; SD: ±3.42)	10.54 (7–16; SD: ±2.48)	0.000 **
Self-Control	12.29 (4–15; SD: ±2.20)	11.87 (8–15; SD: ±1.77)	0.228
General Health	11.28 (0–15; SD: ±2.87)	10.20 (4–15; SD: ±2.78)	0.027 *
Vitality	13.89 (5–20; SD: ±3.10)	12.09 (7–17; SD: ±2.87)	0.001 **

**Table 4:** Abbreviations: SFQ Social Functioning Questionnaire; SD: Standard Deviations; ^a^ Independent *t*-test; * *p*-value < 0.05, ** *p*-value < 0.005.

Number of Cases	NH (95)	SSD (54)	*p* Value ^a^
SFQ	4.4 (2–12; SD: ±2.05)	5.7 (4–9; SD: ±1.3)	<0.0001 **

## Data Availability

Raw data were generated at the Azienda Ospedaliero-Universitaria of Modena. Derived data supporting the findings of this study are available from Daniele Monzani upon request.

## References

[1] Ricketts T., Lindley G., Henry P. (2001). Impact of compression and hearing aid style on directional hearing aid benefit and performance. Ear Hear..

[2] Choi J.S., Wu F., Park S., Friedman R.A., Kari E., Volker C.C.J. (2021). Factors Associated with Unilateral Hearing Loss and Impact on Communication in US Adults. Otolaryngol. Head Neck Surg..

[3] Tyagi A.K., Gupta K., Kumar A., Varshney S., Sood R., Malhotra M., Priya M., Bhardwaj A. (2020). Rare Causes of Unilateral Sensorineural Hearing Loss in Adults: Our Experience. Indian J. Otolaryngol. Head Neck Surg..

[4] Usami S.-I., Kitoh R., Moteki H., Nishio S.-Y., Kitano T., Kobayashi M., Shinagawa J., Yokota Y., Sugiyama K., Watanabe K. (2017). Etiology of single-sided deafness and asymmetrical hearing loss. Acta Otolaryngol..

[5] Kay-Rivest E., Irace A.L., Golub J.S., Svirsky M.A. (2022). Prevalence of Single-Sided Deafness in the United States. Laryngoscope.

[6] Van de Heyning P., Távora-Vieira D., Mertens G., Van Rompaey V., Rajan G.P., Müller J., Hempel J.M., Leander D., Polterauer D., Marx M. (2016). Towards a Unified Testing Framework for Single-Sided Deafness Studies: A Consensus Paper. Audiol. Neurotol..

[7] Falcón Benítez N., Falcón González J.C., Ramos Macías Á., Borkoski Barreiro S., Ramos de Miguel Á. (2021). Cochlear Implants in Single-Sided Deafness. Comparison Between Children and Adult Populations with Post-lingually Acquired Severe to Profound Hearing Loss. Front. Neurol..

[8] Snapp H.A., Ausili S.A. (2020). Hearing with One Ear: Consequences and Treatments for Profound Unilateral Hearing Loss. J. Clin. Med..

[9] Kumpik D.P., King A.J. (2019). A review of the effects of unilateral hearing loss on spatial hearing. Hear. Res..

[10] Gatehouse S., Noble W. (2004). The Speech, Spatial and Qualities of Hearing Scale (SSQ). Int. J. Audiol..

[11] Bess F.H., Davis H., Camarata S., Hornsby B.W.Y. (2020). Listening-Related Fatigue in Children With Unilateral Hearing Loss. Lang. Speech Hear. Serv. Sch..

[12] Lewis D., Schmid K., O’Leary S., Spalding J., Heinrichs-Graham E., High R. (2016). Effects of Noise on Speech Recognition and Listening Effort in Children with Normal Hearing and Children With Mild Bilateral or Unilateral Hearing Loss. J. Speech Lang. Hear. Res..

[13] Härkönen K., Kivekäs I., Rautiainen M., Kotti V., Sivonen V., Vasama J.P. (2015). Single-Sided Deafness: The Effect of Cochlear Implantation on Quality of Life, Quality of Hearing, and Working Performance. ORL J. Otorhinolaryngol. Relat. Spec..

[14] Bess F.H., Tharpe A.M. (1984). Unilateral hearing impairment in children. Pediatrics.

[15] Lucas L., Katiri R., Kitterick P.T. (2018). The psychological and social consequences of single-sided deafness in adulthood. Int. J. Audiol..

[16] Voola M., Távora-Viera D. (2020). Quality of Life handicap measured in patients with profound unilateral or bilateral deafness. Tasman. Med. J..

[17] Sood R., Varshney S., Gupta K., Devi N.S., Kumar N., Tyagi A.K., Kumar A. (2022). The Impact of Unilateral Sensorineural Hearing Loss on Quality of Life of Sub-Himalayan Population. Int. J. Otolaryngol..

[18] Sood R., Gupta K., Varshney S., Kumar A., Tyagi A.K., Devi N.S. (2022). Spectrum of Handicap in Unilateral Sensorineural Hearing Loss. Indian J. Otolaryngol. Head Neck Surg..

[19] Newman C.W., Weinstein B.E., Jacobson G.P., Hug G.A. (1990). The Hearing Handicap Inventory for Adults: Psychometric adequacy and audiometric correlates. Ear Hear..

[20] Monzani D., Genovese E., Palma S., Rovatti V., Borgonzoni M., Martini A. (2007). Measuring the psychosocial consequences of hearing loss in a working adult population: Focus on validity and reliability of the Italian translation of the Hearing Handicap Inventory. Acta Otorhinolaryngol. Ital..

[21] Sanders D.A., Pollack M.C. (1975). Hearing Aid Orientation and Counseling. Amplification for the Hearing-Impaired.

[22] Martini A., Mazzoli M., Rosignoli M., Trevisi P., Maggi S., Enzi G., Crepaldi G. (2001). Hearing in Elderly: A Population Study. Audiology.

[23] Dupuy H.J., Wenger N.K., Mattson M.E., Furberg C.D., Elinson J. (1984). The Psychological General Well-Being (PGWB) Index. Assessment of Quality of Life in Clinical Trials of Cardiovascular Therapies.

[24] Grossi E., Mosconi P., Groth N., Niero M., Apolone G. (2002). Questionario Psychological General Well.-Being Index: Versione Italiana.

[25] Tyrer P., Nur U., Crawford M., Karlson S., MacLean C., Rao B., Johnson T. (2005). The Social Functioning Questionnaire: A Rapid and Robust Measure of Perceived Functioning. Int. J. Soc. Psychiatry.

[26] Zanello A., Arpone F., Colin L., Merlo M.C.G. (2011). Adattamento italiano del Questionario del Funzionamento Sociale (QFS). Riv. Sper. Freniatr..

[27] Galloway J., Zhang V., Marnane V., Hou S., Stewart G., Bardy F. (2019). The impact of unilateral hearing loss on adult life. Hear. Review.

[28] Chang P.F., Zhang F., Schaaf A.J. (2020). Deaf in one ear: Communication and social challenges of patients with single-sided deafness postdiagnosis. Patient Educ. Couns..

[29] Danermark B., Gellerstedt L.C. (2004). Psychosocial work environment, hearing impairment and health. Int. J. Audiol..

[30] Nachtegaal J., Kuik D.J., Anema J.R., Goverts S.T., Festen J.M., Kramer S.E. (2009). Hearing status, need for recovery after work, and psychosocial work characteristics: Results from an internet-based national survey on hearing. Int. J. Audiol..

[31] Kramer S.E., Kapteyn T.S., Houtgast T. (2006). Occupational performance: Comparing normally-hearing and hearing-impaired employees using 61 the Amsterdam Checklist for Hearing and Work. Int. J. Audiol..

[32] Wie O.B., Hugo Pripp A., Tvete O. (2010). Unilateral deafness in adults: Effects on communication and social interaction. Ann. Otol. Rhinol. Laryngol..

[33] Wolber L.E., Pryce H. (2016). Measuring the impact of illness perception on NHS audiology service usage in presbycusis patients—A feasibility study. Hear. Balance Commun..

[34] Chia E.M., Wang J.J., Rochtchina E., Cumming R.R., Newall P., Mitchell P. (2007). Hearing impairment and health-related quality of life: The Blue Mountains Hearing Study. Ear Hear..

[35] Monzani D., Galeazzi G.M., Genovese E., Marrara A., Martini A. (2008). Psychological profile and social behaviour of working adults with mild or moderate hearing loss. Acta Otorhinolaryngol. Ital..

